# Supplementary material for: Are carbon emissions trading and green financial instruments synergistic? -Comprehensive quantitative research based on content analysis
Source: PLoS One. 2024 Mar 7;19(3):e0298601. doi: 10.1371/journal.pone.0298601 (PMC10919601; doi:10.1371/journal.pone.0298601)
Supplement: S1 Data — (ZIP) [file pone.0298601.s001.zip › Data/地级市Z_F工作报告.zip!/╣≤╓▌/┴∙┼╠╦«╩╨/└┤╫╘╣1⁄2╓┌║┼í╛┬φ┐╦╩2╛▌═°í┐_2011.docx]

各位代表：

　　现在，我代表市人民政府，向大会报告工作。根据《中共六盘水市委关于制定六盘水市国民经济和社会发展第十二个五年规划的建议》，市人民政府制定了《六盘水市国民经济和社会发展第十二个五年规划纲要（草案）》，请各位代表连同政府工作报告一并审议，并请各位政协委员和列席的同志提出意见。

　　“十一五”时期国民经济和社会发展回顾

　　过去五年，在省委、省政府和市委的坚强领导下，在市人大、市政协的监督、支持和帮助下，我们坚持以邓小平理论、“三个代表”重要思想为指导，深入贯彻落实科学发展观，坚持“加速工业化、加快城镇化、推进农业产业化”和“统筹城乡、跨越发展”的思路，克服了百年不遇的雪凝灾害、金融危机和特大旱灾的影响，圆满完成了“十一五”规划的各项目标任务，全市经济社会发展取得了巨大成就。

　　──综合经济实力迈上新台阶。全市生产总值由2005年的209亿元增加到2010年的500.64亿元，年均增长15.2%。全社会固定资产投资累计完成1098.48亿元，年均增长22.5%。财政总收入由2005年的30.04亿元增加到2010年的107.89亿元，年均增长29.14%；财政一般预算收入从13.23亿元增加到49.3亿元，年均增长30.09%。社会消费品零售总额从58.06亿元增加到131.34亿元，年均增长17.7%。2010年末金融机构存、贷款余额分别为500.8亿元和361.7亿元，是2005年末的2.55倍和2.59倍。西部大开发战略各项重点工作加快推进，“五年打好基础、十年重点突破”的目标基本实现。

　　──经济结构调整取得新进展。农业综合生产能力稳步提升，粮食生产基本稳定。以马铃薯、蔬菜、畜牧业为重点的种养业规模化、商品化水平不断提高，马铃薯、蔬菜种植面积达200万亩和45万亩，畜牧业产值占农业总产值的比重年均提高1个百分点。各级财政累计投入支农资金40.3亿元，是“十五”的4.58倍；工业主导地位进一步提升，煤炭、电力、冶金、建材等支柱产业不断巩固和壮大，煤化工、装备制造业加快发展，食品加工业、农产品加工业发展取得成效；金融、物流、信息、旅游等服务业呈现出旺盛的发展势头，第三产业增加值年均增长15%。省下达我市节能减排目标顺利完成，万元生产总值能耗降低3.31%，5个县级污水处理厂全部投入试运行，火电机组全部安装脱硫设施并投入运行。加快推进经济发展方式转变，循环经济发展取得积极进展，我市被国务院列为资源富集区开展循环经济区试点。

　　──基础设施建设实现新突破。“十一五”时期，以交通为重点的基础设施建设取得重大突破，完成交通固定资产投资104亿元。六沾铁路复线基本建成；高速公路从无到有，镇胜高速公路境内段建成通车，水盘高速公路、六枝至镇宁高速公路、水城至六枝高速公路、杭瑞高速公路境内段开工建设；建成了一批二级公路、通乡油路、通村公路和运煤公路；六盘水月照机场工可获国家正式批准；建成了毛口、野钟、中寨航运码头。大力破解工程性缺水难题，黔中水利枢纽一期工程和白河沟、双桥、鱼洞坝、旧院等一批重点水利工程开工建设，新增、改善和恢复有效灌溉面积20万亩，解决了52.3万名农村人口的饮水安全问题。加强电力基础设施建设，建成了一批500KV、220KV、110KV输变电站，城乡用电保障能力不断提高。加大生态建设和保护力度，治理石漠化面积134平方公里，全市森林覆盖率达37%，年均提高1个百分点以上。

　　──改革开放取得新成效。国有企业改革脱困工作稳步推进，国有资产监管体系进一步完善。财政改革不断深化，财政预算刚性得到强化，财政国库管理制度全面建立。金融改革步伐加快，实现全市乡镇金融机构全覆盖。投融资体制改革取得新突破，六盘水市商业银行挂牌成立，并实现从地方性银行向区域性银行战略转型，组建了六盘水市商业银行贵阳分行；运用BT、BOT模式建设了一批基础设施。农村综合改革稳步推进，集体林权制度主体改革基本完成。行政管理体制改革扎实推进，市、县政府机构改革全面完成。价格、行政审批制度以及文化体制、医药卫生等改革取得新进展。整顿和规范市场秩序成效明显。非公有制经济不断发展壮大，占生产总值的比重从27.5%提高到36%。对外开放水平进一步提高，区域经济合作和对口帮扶不断深入。累计招商引资实际到位资金312.59亿元，是“十五”的3.14倍。

　　──人民生活水平得到新提高。各级财政共投入民生方面资金达190亿元，是“十五”的4.75倍。城镇居民人均可支配收入和农民人均纯收入分别从2005年的8032元、1863元增加到2010年的14428元、3600元，年均实际增长9%和9.5%。减少农村贫困人口15.6万人。城市低保月人均补助水平从72元提高到136元；全面实施了农村居民最低生活保障制度，年人均保障标准达到1243元，共保障7万余名城镇困难人口和43万余名农村贫困人口。新型农村合作医疗制度实现全覆盖，城镇职工基本医疗、基本养老、失业、工伤和生育保险覆盖面不断扩大，保障水平不断提高。钟山区新型农村养老保险试点工作扎实推进。城镇居民基本医疗保险制度全面推行，医疗救助和临时救助制度实现全覆盖。新建农村敬老院60所。市级财政投入1500万元建立了创业基金，启动了省级创业型城市创建工作。城镇新增就业9.3万人，比“十五”增长17.2%。城镇登记失业率控制在4.5%以内。完成3.6万户农村危房改造和17.22万平方米的经济适用住房建设，发放廉租房租赁补贴5425万元。全面完成国有重点煤矿采煤沉陷区综合治理工程。启动了5200户农村“美好家园”建设试点。投入救灾救济资金1.72亿元，救济灾民317.1万人次。安全生产持续稳定好转，“十一五”期间各项安全考核指标全面完成。扎实推进“平安凉都”建设，群众安全感有所提高。信访和维稳工作进一步加强，全市社会大局保持稳定。

　　──社会事业取得新进步。全面实施城乡免费义务教育，“两基”工作通过国家督导评估检查；全市高中阶段毛入学率从2005年的29.54%提高到47.74%；整合地企职业技术教育资源取得实质性进展，培养了11.5万名中等职业教育毕业生；六盘水高等师范专科学校升格为六盘水师范学院，结束了六盘水市没有本科院校的历史。覆盖城乡的公共卫生和医疗服务体系逐步建立，完成97个乡镇卫生院改扩建，建成881个标准化村卫生室；新型农村合作医疗参合率从71.16%提高到97.71%，筹资标准较“十五”提高100元。建成7887个20户以上已通电自然村广播电视“村村通”工程，全面完成市、县数字电视整体转换平移工作。4个县、特区、区全部通过省“科技进步县”考核。全民健身运动广泛开展。成功举办了5届消夏文化节。文化自然遗产保护工作得到加强。人口出生率从14.45‰下降到12.69‰。妇女、儿童、老龄和残疾人事业取得新进展。民主法制和精神文明建设不断加强，“五五”普法通过省级检查验收，依法行政和廉政建设深入推进，国防建设与经济建设协调发展。民族、宗教、外事、侨务、对台、档案、气象、人防、青少年、工会、红十字等工作取得新成绩。

　　2010年，全市经济实现平稳较快发展，为全面完成“十一五”规划划上了圆满句号。全市生产总值增长15.8%；财政总收入和一般预算收入分别增长28.34%和32.82%；全社会固定资产投资增长34.44%；社会消费品零售总额增长18.67%；城镇居民可支配收入和农民人均纯收入分别实际增长6.5%和13.8%。

　　在历史罕见的挑战和风险面前,过去五年取得的成绩确实来之不易，形成的发展基础、积累的宝贵经验、创造的精神财富必将对六盘水未来发展产生深远影响。实践证明，过去五年是全市改革发展取得重大进展的五年，是综合实力和竞争力大幅提升的五年，是社会事业全面进步和人民群众获得更多实惠的五年。

　　通过“十一五”时期的实践与探索，我们对做好我市工作有以下体会：一是必须坚定不移地深入贯彻落实科学发展观，坚持把中央的路线方针政策与六盘水实际结合起来，积极探索符合六盘水市情、体现六盘水经济社会发展阶段性特征的科学发展之路，不断开创科学发展新局面，在做大经济总量中提高发展质量，在推进跨越中实现全面小康。二是必须坚定不移地用统筹的办法解决发展中的问题，处理好整体推进与重点突破、经济发展与环境保护、政府引导和市场调节等方面的关系，抓住主要矛盾和矛盾的主要方面，重点攻关，推动全局。三是必须坚定不移地推进改革开放，加快重点领域和关键环节改革，构建充满活力、富有效率、更加开放、有利于科学发展的体制机制，积极鼓励大胆试、大胆闯、敢为人先，在解放思想、探索创新中推进发展。四是必须坚定不移地把保障改善民生作为政府工作的出发点和落脚点，更加注重包容性增长和共享式发展，切实做好就业、社保、教育、文化、卫生等工作，让全市人民共享改革发展的成果。

　　各位代表：“十一五”期间我市经济社会发展取得的成绩，为我市“十二五”又好又快、更好更快发展奠定了坚实基础。这些成绩的取得，是党中央、国务院和省委、省政府正确领导，省直各部门大力支持的结果；是全市各级各部门全面贯彻落实科学发展观的结果；是全市各族干部群众抢抓机遇、艰苦奋斗的结果。在此，我代表市人民政府，向在过去五年中为六盘水市经济社会发展作出艰苦努力和付出辛勤劳动的全市广大工人、农民、知识分子、干部，向各民主党派、工商联、无党派爱国人士和各人民团体，向驻市人民解放军、武警官兵、公安干警、民兵和预备役人员，向所有关心和支持我市改革开放和现代化建设的各界人士，表示衷心的感谢并致以崇高的敬意！

　　在肯定成绩的同时，我们也清醒地看到发展中存在的矛盾和问题，主要是：经济总量小，人均水平低，发展速度不够快；城乡二元结构突出，农村贫困程度深，农民收入增长缓慢；资源依赖型经济结构和粗放型发展方式尚未根本改变，资源综合利用水平不高，节能减排压力较大；经济与社会发展协调性还不够；政府自身建设还存在薄弱环节。对于这些矛盾和问题，我们要高度重视，采取有力措施加以解决。

　　“十二五”时期国民经济和社会发展的主要目标和任务

　　“十二五”时期，是我市可以紧紧抓住并且大有作为的战略机遇期，也是全面建设小康社会承上启下的关键期。综观国内外形势，机遇与挑战并存，机遇大于挑战，总体上有利于我们进一步加快发展。国家扩大内需的方针，深入实施西部大开发战略，中央企业加速扩张，东部产业加快转移，有利于我们争取中央支持、借助外力加速发展；经过长期艰苦奋斗，我市发展的基础条件明显改善，产业基础更加坚实，人均生产总值突破2500美元，城镇化率达到35%，正进入工业化和城镇化加速发展的新阶段，有利于我们谋求更好更快发展。发展态势表明，面向未来，我们站在了实现六盘水历史性跨越的新起点上。只要我们科学把握发展规律，主动适应新形势，积极抢抓新机遇，勇于迎接新挑战，加倍努力踏实工作，就会在新的起点上推动经济社会继续向前发展，就能够开创六盘水更加美好的未来。

　　“十二五”时期，我市经济社会发展的指导思想是：高举中国特色社会主义伟大旗帜，以邓小平理论和“三个代表”重要思想为指导，深入贯彻落实科学发展观，解放思想，更新观念，抢抓国家深入实施西部大开发战略和国务院将六盘水市列为资源富集区开展循环经济区试点等机遇，以科学发展为主题，以转变经济发展方式为主线，以“加速发展、加快转型、推动跨越”为主基调，以加大投资和项目建设为抓手，大力实施工业强市和城镇化带动战略，着力推进农业产业化，发展壮大特色优势产业，进一步改善基础设施，加快社会事业发展，深入推进改革开放，切实保障改善民生，大力促进社会和谐，把六盘水建成产业聚集的现代工业城市、交通便捷的枢纽城市、宜业宜居的生态人文城市，使“十二五”成为六盘水建市以来发展最好最快的时期之一，为实现到2020年与全国同步进入全面小康社会打下坚实基础。

　　“十二五”时期，我市经济社会发展的主要预期目标是：到2015年，全市生产总值达到1200亿元以上，在2010年的基础上翻一番以上，年均增长16%以上。人均生产总值达到5500美元；全社会固定资产投资五年累计达4000亿元左右，年均增长30%以上；财政总收入达270亿元，一般预算收入达110亿元；社会消费品零售总额达300亿元，年均增长16%以上；城镇居民人均可支配收入和农民人均纯收入分别年均增长12%以上和13%以上；城镇化率达到50%以上；人口出生率控制在13‰以内；城镇登记失业率控制在4.5%以内；森林覆盖率达45%；完成省下达节能减排指标。

　　关于“十二五”时期我市经济社会发展的工作和政策措施，《六盘水市国民经济和社会发展第十二个五年规划纲要（草案）》已作了部署，这次会议审议通过后，我们将认真抓好落实。今后五年，重点抓好以下六个方面工作。

　　（一）大力实施工业强市战略，走新型工业化发展道路。立足我市资源优势和工业基础，按照在扩大总量中调整优化结构、实现工业转型升级的要求，继续做大做强煤炭、电力、冶金、建材产业，加快发展煤化工、装备制造业，提高对工业增长的支撑能力；加快培育高新技术产业、特色农产品加工业、医药工业、特色旅游商品业等产业，促进工业结构的高新化、轻型化。大力推进产业园区建设，力争建成一批国家级、省级重点工业园区，促进产业集群、集聚、集约发展。通过大力实施工业强市战略，把我市建成全国循环经济示范区和西南地区重要的能源基地、资源深加工基地、装备制造业基地。“十二五”时期，全市工业固定资产投资累计完成2500亿元以上，力争达3000亿元，工业增加值年均增长22%以上，规模以上工业企业超过1000户，建成一批销售收入超过100亿元的大企业、大集团，培育一批上市公司。

　　（二）大力实施城镇化带动战略，增强城镇对经济社会发展的辐射带动能力。遵循城镇化发展规律，根据人口、资源、环境和经济社会发展的需要和可能，充分发挥大中城市的辐射带动作用，促进大中小城市和小城镇的协调发展，走有特色、集约型、多样化的山区城镇化道路。通过拉大城市框架、推进组团发展、完善城市功能、强化产业支撑，把市中心城区建设成为80万人口以上的大城市，力争到2020年建设成为120万人口以上的特大城市；盘县红果向大城市、六枝平寨向中等城市目标迈进；结合二、三产业发展，大力培育一批层次分明、布局合理、功能互补的小城市和中心镇。加大城市基础设施和公共配套设施建设力度，深入推进“双创双建”活动，大力发展旅游、金融、物流、信息等现代服务业，不断提高城市综合承载能力。深化户籍管理制度改革，改善农民工就业、居住、就医和子女就学等基本生活条件，加快农业人口向城镇梯度转移。“十二五”末，城镇人口人均道路面积达8平方米以上，建成区绿地率达到30%，城镇用水普及率达95%，城镇人口人均住房面积达28平方米以上。

　　（三）加快农业结构调整，推进农业产业化发展。坚持以“统筹城乡、跨越发展”总揽“三农”工作全局，在工业化、城镇化加速发展中，同步推进农业产业化发展，促进农业增效、农民增收。认真落实各项强农惠农政策，建立财政支农投入的稳定增长机制，引导各类社会资金投向“三农”。稳步提高农业综合生产能力，到2015年建成农业人口人均0.5亩高产稳产基本口粮田。大力调整农业结构，加快发展农业优势特色产业。马铃薯产业在稳定现有种植面积的基础上，重点在脱毒种薯繁育基地建设、高产示范基地建设、加工和市场经营上取得新突破，把六盘水建成贵州重要的马铃薯商品生产基地及出口基地；畜牧业以推进规模养殖场（小区）建设、扶持养殖大户为突破口，大力发展草地生态畜牧业，力争畜牧业产值占农业总产值的比重达42%以上；蔬菜产业以扩大城郊商品蔬菜基地规模为重点，在丰富品种、提高单产、改善品质、增加效益上下功夫，确保3年内实现大宗蔬菜自给有余。积极培育和引进农业产业化经营龙头企业，加快发展特色农产品加工业。大力扶持农民专业合作经济组织，完善企业与农民的利益联结机制。加强农村劳动力技能就业培训，大力发展劳务经济。实施水、电、气、路、房、优美环境“六到农家”工程，加快建设农村“美好家园”。扎实推进新阶段扶贫开发。到2015年，农业总产值达100亿元以上，农民人均纯收入达7000元；按照国家扶贫标准，农村贫困人口数量比2010年减少一半。

　　（四）加强基础设施建设，不断改善经济社会发展的基础条件。加快推进以快速铁路、高速公路、支线机场等为重点的交通基础设施建设，形成连通内外、覆盖城乡的综合交通网络。积极争取国家支持，加快推进毕水兴、水城至攀枝花等一批对经济社会发展有重要带动作用的铁路及货场建设。建成杭瑞高速公路境内段、镇宁—六枝—水城—威宁高速公路、水盘高速公路，开工建设市中心城区外环高速西环线、纳雍—六枝—晴隆高速公路，基本形成“两横两纵一环线”高速公路主骨架。建成一批二级公路、通乡油路、通村公路和运煤公路，到2015年实现70%以上的行政村通油路或水泥路。进一步健全公路管理养护体制机制，实现有路必养、养必到位。建成中寨二期、格支、格所等水运码头，完善北盘江航运基础设施。建成六盘水月照机场。加快水利发展改革，把水利作为基础设施建设的优先领域，把农田水利作为农村基础建设的重点任务，把严格水资源管理作为加快转变经济发展方式的战略举措，突出加强薄弱环节，大力发展民生水利。紧紧抓住国家编制贵州省水利建设、生态建设、石漠化治理“三位一体”规划和加强中小河流治理及山洪地质灾害防治的机遇，新建一批重点水利工程。配合抓好黔中水利枢纽工程，建成白河沟、渔洞坝、旧院、双桥等水库，开工建设卡河水库，进一步完善农业、农村和城市生产、生活用水工程体系。到2015年，全面解决农村饮水安全问题，初步达到城乡经济社会发展供水、饮水、防洪、水生态环境“四大安全”目标。加快电力基础设施建设，建成一批500KV、220KV和110KV的输变电工程，提升供电可靠性和供电能力。加快信息基础设施建设。

　　（五）深化改革和扩大开放，努力营造良好的发展环境。继续深化重点领域和关键环节体制改革。全面推进国有企业改革改制。深入推进财政体制改革，完善事权财权相匹配的财政体制。深化金融体制改革，积极发展中小金融机构和小额贷款公司。深化投资体制改革，切实加强监管，提高投资效益。大力推进企业上市融资，力争“十二五”期间新增一批上市公司。加强融资性担保机构体系建设，积极引导和鼓励民间资本投向“十二五”规划重点产业。深化农村改革，做好集体林权制度配套改革工作，推进农村土地承包经营权有序流转，积极开展农村宅基地使用权抵押贷款工作。积极推进社会保障和教育、文化、卫生等社会事业领域体制改革；扩大对内对外开放。积极开展全方位、多层次区域合作，在更大的范围内布局我市生产力，主动融入成渝、黔中、西江上游、滇中等经济区，积极构建“攀西—六盘水”经济区；着力抓好招商引资工作。充分利用我市资源优势，以产业园区为载体，抓住国际、国内产业梯度转移的有利时机，充实和完善招商引资项目库，大力改善发展环境，积极引进中央企业、东部沿海企业和世界500强、国内500强企业到六盘水投资，借力、借势、借梯发展；大力支持非公有制经济加快发展。坚持“两个毫不动摇”，认真贯彻落实中央、省、市鼓励非公有制经济发展的各项政策措施，为非公有制经济创造优良的发展环境，到“十二五”末，力争非公有制经济占生产总值比重提高到40%以上。

　　（六）大力保障和改善民生，推进和谐社会建设。统筹抓好城乡就业，建立健全促进就业的长效机制，千方百计扩大就业；大力推动创业带动就业，争取成为国家级创业型试点城市。切实抓好城乡低保，完善基本养老、基本医疗、生育、失业、工伤等保障体系，健全社会救助体系，构建住房保障体系,加快建立覆盖城乡居民的社会保障体系。加快科技、教育、卫生、文化、体育等各项社会事业发展。加大科技创新投入，加强创新载体建设，加快科技成果转化。在巩固“两基”攻坚成果的同时，重点加强高中阶段教育、职业教育和高等教育，到2015年，市中心城区及县城所在地城区3—6岁幼儿入园率和高中阶段毛入学率分别达到90%和75%。加强公共卫生和医疗服务体系建设，逐步建立覆盖城乡居民的基本医疗卫生制度，努力满足人民群众医疗卫生服务需求。加强文化自然遗产保护。加强社会建设和管理。深入开展“平安凉都”创建活动。加强和改进信访工作。加大公共安全投入，健全预防预警和应急处置体系,提升各行业、各领域安全生产水平。加强对民族地区的扶持，加快少数民族群众脱贫致富步伐。加强社会主义精神文明和民主法治建设。强化国防建设。切实做好老龄、妇女、儿童、青少年、工会、残疾人等工作。

　　2011年经济社会发展的主要目标和任务

　　今年是深入实施西部大开发战略第二个十年的第一年，是实施“十二五”规划的起步之年，又是我们党建党90周年。做好今年的政府工作，对“十二五”开好局、起好步，意义十分重大。全市经济社会发展的主要预期目标是：生产总值增长16%以上；全社会固定资产投资增长35%以上；财政总收入、一般预算收入增长20%以上；规模以上工业增加值增长22%以上；社会消费品零售总额增长18%以上；城镇居民人均可支配收入和农民人均纯收入分别增长10%；城镇登记失业率控制在4.5%以内；人口出生率控制在12.8‰以内；完成省下达的节能减排指标。

　　围绕上述目标，我们将着力做好以下七个方面工作。

　　(一)工业化方面。一是推进“六大支柱产业”发展壮大。煤炭产业，加大煤矿整合力度，加快建成一批新矿井和技改矿井，全市煤炭产量力争达6400万吨。电力产业，尽快开工建设六枝电厂、盘县电厂“上大压小”、盘北和汪家寨煤矸石发电厂等重点火电项目，全年发电量突破350亿度。煤化工产业，大力抓好老鹰山煤化工二期、黔桂天能130万吨煤焦化扩建、玉舍循环经济煤焦化、红果恒鼎循环经济煤焦化等一批新型煤化工项目的实施。统筹推进煤层气、焦炉煤气、过境天然气的综合开发利用。冶金产业，支持首钢水城钢铁（集团）公司全面完成扩能改造，加快推进首黔新工艺新材料循环经济工业示范基地建设，年内形成500万吨钢的产能，钢材产量突破400万吨。加快推进铝工业优化升级，开工建设高压电子铝箔加工项目。建材产业，加快推进已核准的新型干法水泥熟料生产线建设，支持鼓励以大宗工业废弃物为原料发展新型墙体材料，全年水泥产量突破600万吨。装备制造业，大力支持境内企业做大做强，积极支持中煤科工、中机工业、北方重工等企业到我市投资建厂，努力在煤矿成套装备、铸造加工、重型汽车及零部件等重大装备技术及产品上实现新突破。在抓好六大产业的同时，因地制宜地加快培育高新技术产业、特色农产品加工业、医药工业、特色旅游商品业等产业。二是强力推进园区建设。高标准、高起点编制园区各项规划，启动建设各级各类园区。建立健全管理体制机制，配强干部，充实人员。扎实开展产业项目前期工作，审批和开工建设一批产业项目。今年，重点产业园区建设要确保完成投资500亿元以上。三是加强企业挖潜改造。积极支持和鼓励企业开展自主创新、发明创造，积极推广使用新技术、新工艺、新装备，加强与科研院所、大专院校、先进工业企业、同行企业在技术上的对接与合作，走技术创新和内涵式发展的道路。四是支持中小企业加快发展。切实为中小企业搞好服务，帮助企业完善法人治理结构、搞好人才培养。坚持“非禁即允”和平等待遇的原则，为非公有制企业发展创造更加宽松的环境，大力推进非公有制经济更好更快发展。

　　（二）城镇化方面。一是优化城镇空间布局。按照组团式布局、集群化发展的要求，超前布局全市城镇体系。加快推进“老鹰山—滥坝”、“汪家寨—大河”、“玉舍—勺米”等组团发展，不断拓展市中心城区范围，大力推进大湾、木果、南开、化乐等卫星城镇建设；加快推进盘县红果与城关、火铺、西冲、刘官、两河等乡镇，六枝平寨与新窑、大用、落别、木岗等乡镇一体化发展。高度重视城乡规划编制工作，加大城市控制性详细规划和城镇、乡村所在地建设规划的编制力度，力争达到全覆盖。完成市中心城区综合交通、住房建设、城市风貌等一批专项规划。二是加快提升城镇综合承载能力。以路网建设为重点，同步配套建设水、电、气、通信、污水、垃圾处理等市政基础设施和医院、学校等公益服务设施。重点抓好凤凰山片区开发、水城河综合治理、老城改造和明湖湿地公园建设；进一步完善城市路网，开工建设乌蒙大道、双龙路、水西南路、凤凰大道东段、水钢西出口道路等工程，启动市中心城区到“老鹰山—滥坝”、“汪家寨—大河”、“玉舍—勺米”等组团的快速通道建设前期工作。积极发展以旅游业为龙头的服务业，着力改造提升传统服务业，大力发展现代服务业。加快推进旧城改造和“城中村”改造。加强社区建设和管理，大力发展社区服务业。强力推进“双创双建”工作。组织实施“数字六盘水”建设规划，建成市中心城区数字化城管一期工程。三是创新城镇发展机制。加强土地运作，启动土地“收购—储备—整理—拍卖—开发”；认真清理处置闲置土地；推行净地出让和先安置、后拆迁。进一步探索经营城市的路子，采取出让城市道路桥梁冠名权、拍卖广告权、有选择地出让部分市政设施的使用权和经营权等多种方式，多渠道筹集城市建设资金。四是加快推进农村人口向城镇转移。建立支持农民转户进城政策体系，做到“两换两留一同”：即允许农民以宅基地（住房）换公租房或经济适用住房，允许农民以承包地换养老保险、医疗保险和城市低保；农民转户后继续保留林地使用权，5年内保留对农村计划生育政策的享受；转户后农民工在就业和子女入学方面与城镇居民享受同等待遇。

　　（三）“三农”方面。今年重点在“调整、扩大、提升”上下功夫，促进农民增收。一是调整结构。在稳定粮食生产、确保增长5%以上（含恢复性增长）的前提下，充分发挥我市的比较优势，大力发展农业特色产业，坚定不移地抓好马铃薯、畜牧、蔬菜、茶叶、果品和烤烟“六大产业”，积极发展生姜、苦荞、芸豆、中药材等地方特色农产品。大力实施种植业结构战略性调整，启动实施“陡坡地种植玉米退耕计划”。二是扩大规模。组建马铃薯、生态畜牧和商品蔬菜产业发展的工作机构，并增加专项资金投入。今年，马铃薯种植面积要稳定在200万亩以上，建成30万亩马铃薯商品基地，力争马铃薯商品率达到45%以上。加快水城县、盘县马铃薯快繁中心建设；加大优质肉牛、肉羊、肉猪、肉禽基地和良种推广项目建设，实施好省、市确定的重点生态畜牧项目，实现畜牧业产值占农业总产值的比重提高1个百分点以上；扩大蔬菜种植面积，提高蔬菜种植水平，并根据市场需求积极发展反季节蔬菜、无公害蔬菜，增加城市蔬菜自给量，逐步提高蔬菜外销量。积极引进、培育和扶持一批农业产业化经营龙头企业，加快农产品市场体系和农产品加工园区建设。大力发展农村合作经济组织，积极探索农村股份合作制，提高农民的组织化程度。今年，新增2个省级农业龙头企业、4个以上市级龙头企业。三是提升能力。以农田水利建设为重点，加快农村人均0.5亩基本口粮田建设，不断改善农业生产条件，提高抵御自然灾害的能力；继续实施“阳光工程”、“绿色证书”、“雨露计划”等培训，开展引导性培训和职业技能培训1.5万人，农民适用技术培训1万人，提升农民就业技能和种养水平。在加大培训力度的同时，加快农村富余劳动力转移，大力发展劳务经济，切实提高农民的工资性收入；以发展现代农业为目标，加快推进农村土地流转，大力支持龙头企业建设标准化生产基地，积极发展一批现代农业产业园区。今年，在六枝郎岱、水城蟠龙等有条件的地方，启动现代农业产业园区建设。

　　（四）基础设施建设方面。加快推进以交通、水利、电网为重点的基础设施建设，进一步夯实发展基础。配合抓好六沾铁路复线、长昆客运专线境内段和火车站站房改造工程建设，开工建设发嘎坡货场；加快杭瑞高速公路境内段、六枝至镇宁高速公路建设步伐、力争早日实质性开工建设水城至六枝高速公路，力争水盘高速公路年底建成通车。大力推进市中心城区外环高速公路、盘县至兴义高速公路、纳雍—六枝—晴隆高速公路境内段前期工作，启动水城—赫章高速公路前期工作。加快推进英武至大山等一批二级公路建设，全面完成第二、三批运煤公路建设，力争开工建设第四批运煤公路。启动实施“十二五”通村油路建设规划。正式动工建设六盘水月照机场。继续完善北盘江航运基础设施；配合抓好黔中水利枢纽一期工程建设，加快推进白河沟供水工程、双桥水库、鱼洞坝水库、旧院水库建设，力争开工建设卡河水库。积极推进六盘水续建配套及节水改造项目建设。继续推进农村饮水安全工程建设。完成烟水配套工程4.7万亩。治理病险水库8座。积极争取国家小流域治理政策的支持，开工建设一批小流域治理项目。加强水文水资源管理；全面实施新一轮农村电网改造升级。推进一批输变电工程建设进度。加快数字化变电站建设，力争年内建成投运盘县110KV东湖首座数字化变电站。完成鸡场坪、发耳2个集控中心建设。

　　（五）生态建设方面。一是大力实施以石漠化综合治理为重点的生态修复工程和以林业、水土保持为重点的生态建设工程，完成营造林任务12万亩以上，确保森林覆盖率提高1个百分点以上。加强地质遗产保护、地质灾害防治和矿山环境恢复治理。二是大力发展循环经济，重点做好循环经济规划的编制，争取国家批准实施。认真梳理、归纳国家有关支持发展循环经济的政策，并结合我市实际，构建具有六盘水特色的循环经济政策体系。三是切实搞好节能减排，抓好年耗能5000吨标煤以上重点用能单位节能监管，实施重点节能工程，推进建筑、交通、公共机构等重点领域的节能降耗，加大节能产品和技术推广力度；加强全市6个污水处理厂正常运转的监控，严格执行国家产业政策和行业准入标准，加快淘汰高耗能、高排放、低效益的落后生产力，确保企业主要污染物稳定达标排放。

　　（六）改革开放方面。一是深化重点领域改革。坚持以公司制、股份制改革和完善法人治理结构为重点，继续推进国有企业改革改制工作，增强国有企业发展后劲。加强国有资产监管，确保国有资产保值增值。深入推进财税体制改革，进一步理顺并界定上下级政府间财政关系；完善政府预算体系，建立健全规范的预算管理机制。大力推进投融资体制改革，继续把扩大投资作为保持经济平稳较快发展的重要抓手，坚持基础项目、产业项目、民生项目全面推进，国有投资、民营投资、外商投资一起扩大，做好国家政策投资领域和重点的对接工作，强力推进项目建设，千方百计扩大投资规模；深化政银合作、银企合作，积极协助驻市金融机构争取更大的审批和业务创新权限，努力拓宽融资渠道。积极推动交通银行进驻六盘水、中国银行在盘县设立分支机构。完善农村综合改革。扎实推进集体林权制度配套改革。加快供销合作社改革发展。继续深化文化、医药卫生体制等改革。二是实行全方位招商引资。把招商引资作为政府的重要工作，建立领导干部招商引资责任制，制定领导干部带头招商引资考核办法，严格兑现奖惩。创新招商引资方式，推行专业队伍招商，探索组团招商、上门招商等方式，提高招商引资成功率。搭建招商引资平台，以消夏文化节为载体，举办投资贸易洽谈活动，吸引外地客商来我市投资创业。积极参加全国各种投资贸易洽谈和产品展销活动，参加省组织的各种招商引资活动。

　　（七）保障改善民生方面。从今年开始启动实施“十大民生工程”，今年的具体任务是：

　　扶贫攻坚工程。坚持开发式扶贫方针和“大扶贫”格局。全市减少农村贫困人口4.27万人，继续实施好六枝特区陇脚乡、盘县四格乡、水城县玉舍乡和新增六枝特区梭戛乡、水城县金盆乡、杨梅乡“整乡推进、连片开发”，培训转移4300名农村贫困地区劳动力。

　　就业和创业工程。新增城镇就业2.8万人以上，促进下岗失业人员实现再就业2300人以上，帮助1500名就业困难人员实现就业，农业劳动力转移就业5万人。招募不低于100名高校毕业生从事支农、支医、支教、扶贫工作，吸纳200名左右高校毕业生参加就业见习；扶持10个以上高校毕业生创业项目，为100名以上高校毕业生提供创业辅导和支持，组织200名以上高校毕业生参加创业培训工程；建立1个就业见习基地。推进“省级创业型城市”的创建工作；实施“万户小老板创业工程”，增加200户以上小老板，新增就业岗位1000个以上。

　　劳动力素质提升工程。完成100个中小学校校舍安全项目、3个农村义务教育阶段薄弱学校改造工程。完成中等职业技术学校招生15000人以上，培养输送2500名技能人才。新建20所农村幼儿园，每个县城所在地启动建设1所公办幼儿园。启动4个县（特区、区）各3000人规模的普通高中学校建设。启动实施“解决市中心城区大班额问题行动计划”。规范和加强民办教育管理。加快六盘水师范学院建设进度，启动学院二期工程。开工建设六盘水电大双水新校区教学大楼、行政楼。建成并投入使用六盘水职业技术学院图书信息楼、2号综合楼。以提高科技素质、职业技能和经营能力为核心，统筹培训城乡劳动力15000人以上。

　　城乡社会保障体系建设工程。继续扩大城镇职工基本养老、基本医疗、失业、工伤、生育等保险的覆盖面，确保参保人数分别达6.6万人、63万人、9万人、19.2万人和8.7万人。完善城乡居民最低生活保障制度，动态地实现应保尽保。扎实推进钟山区、六枝特区新型农村养老保险试点工作。完善被征地农民社会保障制度，尽快实现与新型农村养老保险试点和城镇职工基本养老保险的制度衔接。规范完善临时救助制度、城乡医疗救助制度，将临时救助资金、农村五保供养资金和农村五保供养机构建设资金纳入财政预算并同比有所提高。

　　保障性住房建设工程。全面完成4.9万户农村危房改造，新开工建设廉租住房14300套、公共租赁住房2200套,改造棚户区11400套，基本解决人均住房建筑面积在15平方米以下的城镇低收入家庭住房问题。启动实施农村教师周转房建设工程和中小学教师安居工程。

　　基础设施向下延伸工程。完成170公里通村油路建设。新增解决13万名农村人口饮水安全问题；治理水土流失面积50.24平方公里；实施中小河流治理工程3个。加强城乡电网和信息基础设施建设，行政村电网完全改造率达100%，农村电网供电可靠率超过98%；完成国家下达的20户以上已通电自然村广播电视“村村通”建设任务和34个乡镇调频(应急)广播工程,大力推进乡镇数字电视整体转换和盘县数字电视双向网改造;新建移动通信基站228个，新增60个自然村通电话。

　　农村生活环境改善工程。开展农村污水、垃圾处理和面源污染治理，完成11个村庄整治和1个农村清洁工程示范点建设任务。建设农村户用沼气1.2万户。建设“美好家园”工程7000户。实施“万村千乡”和“双百市场工程”，完成500个以上农家店、10个以上农贸市场建设。

　　公共文化服务体系建设工程。开工建设市博物馆、图书馆、凤凰山文化广场，继续办好凉都消夏文化节。完成300个农家书屋建设、2个社区文化中心和10个社区文化活动室设备配置，启动2个县级图书馆（文化馆）维修改造、32个乡镇综合文化站设备配置、新建2个县级档案馆；建成6个村级农民体育健身工程、3个乡镇农民体育健身工程、8个社区全民健身路径工程。完成13000场以上农村公益电影放映，文化下乡演出50场次以上。

　　公共卫生事业建设工程。人均公共卫生服务经费达到25元。巩固提高新型农村合作医疗水平，参合率稳定在97%以上。建成并投入使用市人民医院住院综合大楼和市二医外科大楼。开工建设市妇女儿童医院新建住院楼主体工程、市传染病院、市精神病院、市中医院、市中心血站。市人民医院创建三级乙等医院。支持水城矿业（集团）公司总医院、盘江投资控股（集团）公司总医院改扩建及水城钢铁（集团）公司总医院整体搬迁。实施标准化村卫生室建设，实现村村有标准化卫生室。抓好传染病防控和妇幼保健工作。

　　社会管理创新和平安创建工程。进一步完善“大调解”工作格局和机制，建立市、县（特区、区）矛盾纠纷大调解中心，加强乡镇、社区综治工作中心和便民中心的规范化建设。在市、县（特区、区）建立联合接访中心，启动建设“三级视频联动接访”系统。深入开展“平安凉都”创建工作。加强城乡社区警务建设。完善报警与监控系统，建成10000个视频监控点，其中市中心城区建成6000个，实现与省、市、县三级公安机关监控报警联网共享。启动实施“六五”普法规划。加强校园安全管理。毫不松懈地抓好安全生产工作，加强安全生产基层基础，严格落实安全生产责任制，深入开展煤矿、非煤矿山和道路交通等重点领域安全生产专项整治，坚决遏制重特大事故发生，完成省下达的安全生产控制指标。强化食品药品质量监管，保障人民群众健康安全。切实抓好防灾减灾救灾工作，开工建设1个省级救灾物资代储点。

　　加快发展各项社会事业。进一步加强人口和计划生育工作，稳定低生育水平，大力提高人口素质，努力遏制出生人口性别比偏高势头；加强流动人口计划生育管理和服务；新建40个村级计生服务室。完成国家数字测震台、大中城市地震应急指挥中心及国家级地震信息结点建设工作。加强养老服务基础设施建设，积极应对人口老龄化。深入开展全民健身活动，支持发展具有优势和特色的竞技体育。加强“双拥”工作，巩固和发展军政军民团结。加强对妇女儿童发展规划终期监测评估，做好“十二五”妇女儿童发展规划编制工作。推进残疾人事业发展。不断加强人事人才工作。继续做好民族、宗教、侨务、对台、档案、工会、青少年、地方志等工作。

　　加强政府自身建设

　　当前，复杂多变的国内外经济形势、稍纵即逝的发展机遇和全市干部群众盼发展的强烈愿望，对做好政府工作提出了新的更高的要求。我们将按照“团结、务实、勤奋、廉洁”的要求，以“创先争优”和“三个建设年”、“四帮四促”活动为重要抓手，不断加强政府自身建设，为加速发展、加快转型、推动跨越提供有力保障。

　　第一，解放思想，敢闯敢干，做到为政有绩。始终把解放思想作为政府自身建设的先导，破除“等、靠、要”的思想，树立时不我待、抢抓机遇的观念；破除重点不突出、工作一般化的守摊思想，树立勇于开拓、奋发有为的观念；破除求稳怕乱、怕担责任的思想，树立敢抓敢管、敢于碰硬的观念。凡是符合六盘水实际的做法，都大胆创新；凡是外地成功的经验，都大胆借鉴；凡是不利于科学发展的条条框框，都大胆突破，做到先干不争论、先试不议论、先做不评论，在干中积累经验、在干中完善政策。通过解放思想，调动各方面的积极性，在全市上下形成大干快上的氛围。

　　第二，转变作风，狠抓落实，做到从政有力。牢固树立正确的政绩观，大力弘扬雷厉风行、埋头实干的作风，真正把心思放在干事业上、把本领用在促发展上、把功夫下在抓落实上。坚持问政于民、问需于民、问计于民，深入基层、深入群众，满腔热忱地为民履职、帮民解忧，真正做到常怀为民之心、常思为民之策、常行为民之举。大力精简会议和文件，改进会风文风，腾出更多的精力抓工作、办实事、解难题。严格实行问责制，杜绝不作为、摒弃慢作为、惩治乱作为，确保政令畅通，提高政府公信力和执行力。

　　第三，转变职能，提高效率，做到施政有为。深化行政管理体制改革，注重改进政府经济调节和市场监管方式，重视加强社会管理和公共服务，把公共资源更多地放到促进社会统筹发展和提高人民生活水平上。深化行政审批制度改革，该取消的坚决取消，能下放的尽快下放，进一步减少行政审批事项，缩短审批时间，提高运行效率。加强政府机关效能建设，对该办的事坚决办，能办的事马上办，难办的事想法办，涉及多部门的事协调办，努力推进政府工作提速创优。深化政务、村务、厂务公开和公共企业、事业单位办事公开。加强电子政务建设，扩大政府信息公开。

　　第四，科学决策，依法行政，做到执政有章。进一步完善群众参与、专家认证和集体决策相结合的决策机制，促进依法科学民主决策。严格按照法定权限和程序行使权力、履行职责，规范行政行为，做到严格执法、公正执法、文明执法。自觉接受人大及其常委会的法律监督、工作监督和政协的民主监督，广泛听取民主党派、工商联和无党派爱国人士的意见、建议，充分发挥工会、共青团、妇联等人民团体的桥梁纽带作用，自觉接受新闻舆论和社会公众监督，充分保障人民群众的知情权、参与权、表达权、监督权。

　　第五，从严治政，廉洁自律，做到廉政有方。推进惩治预防腐败体系建设，强化对政府公务人员的理想信念和廉洁自律教育。加强领导干部经济责任审计，完善政府重大投资项目公示制和责任追究制，加强对土地管理、工程建设、环境保护等重点领域的行政监察和审计监督，坚决纠正损害群众利益的不正之风，严肃查处各类违法违纪案件。牢固树立过紧日子的思想，按照节俭、高效、廉洁的原则，深入推动节约型机关建设，切实把有限的资金资源更多地用在科学发展和改善民生上。

　　各位代表！蓝图已经绘就，发展充满希望！让我们紧密团结在以胡锦涛同志为总书记的党中央周围，全面贯彻落实党的十七届五中全会、省委十届十次全会、市委五届七次全会精神，在市委的领导下，高举发展、团结、奋斗的旗帜，开拓创新，扎实工作，为全面完成国民经济和社会发展第十二个五年规划、加快推进六盘水经济社会发展的历史性跨越而努力奋斗！
